# Supplementary figures and images for: Effects of Low-Level Deuterium Enrichment on Bacterial Growth
Source: PLoS One. 2014 Jul 17;9(7):e102071. doi: 10.1371/journal.pone.0102071 (PMC4102507; doi:10.1371/journal.pone.0102071)

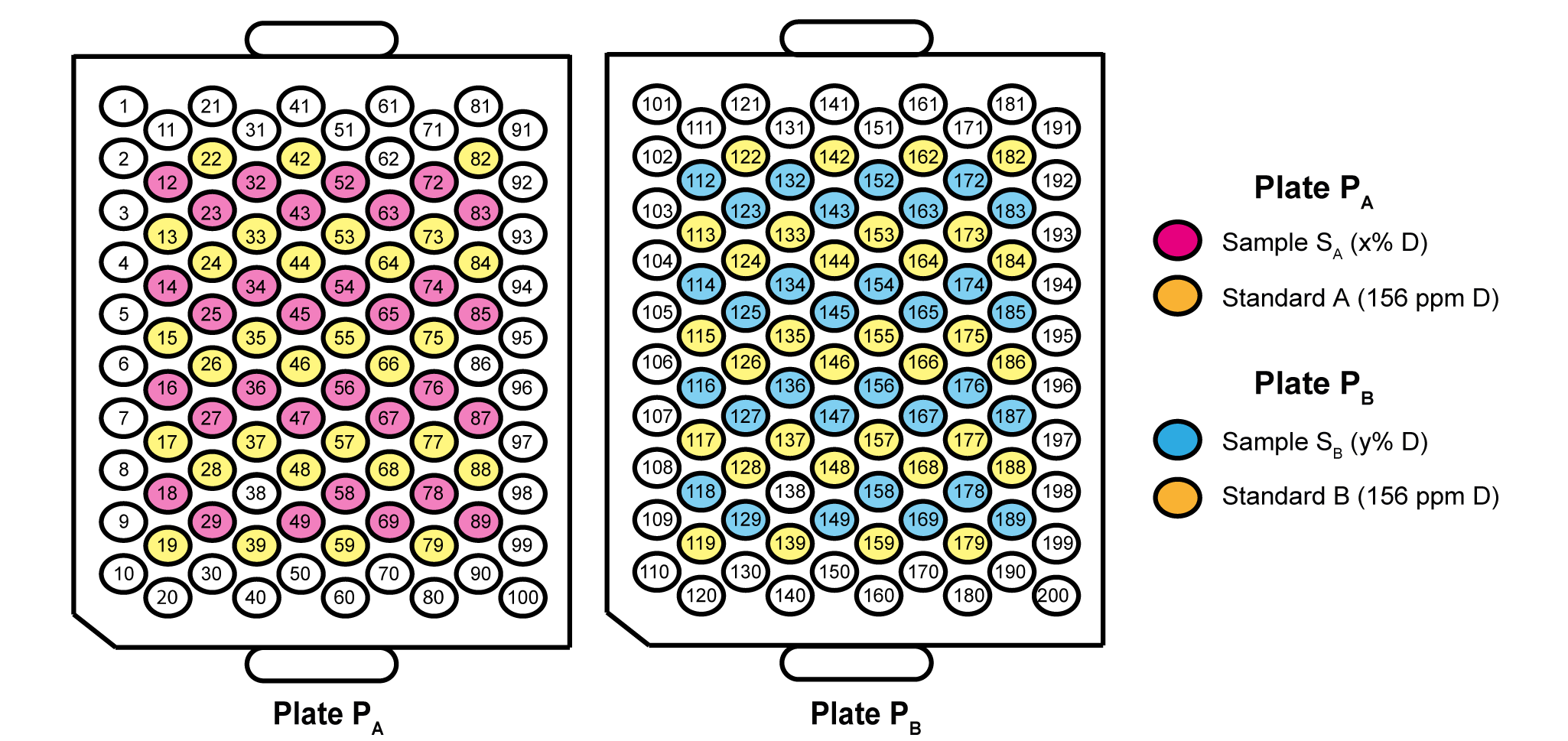

Supplement: Figure S1 — Sample configuration on the honey comb well plates. (TIF) [file pone.0102071.s001.tif]

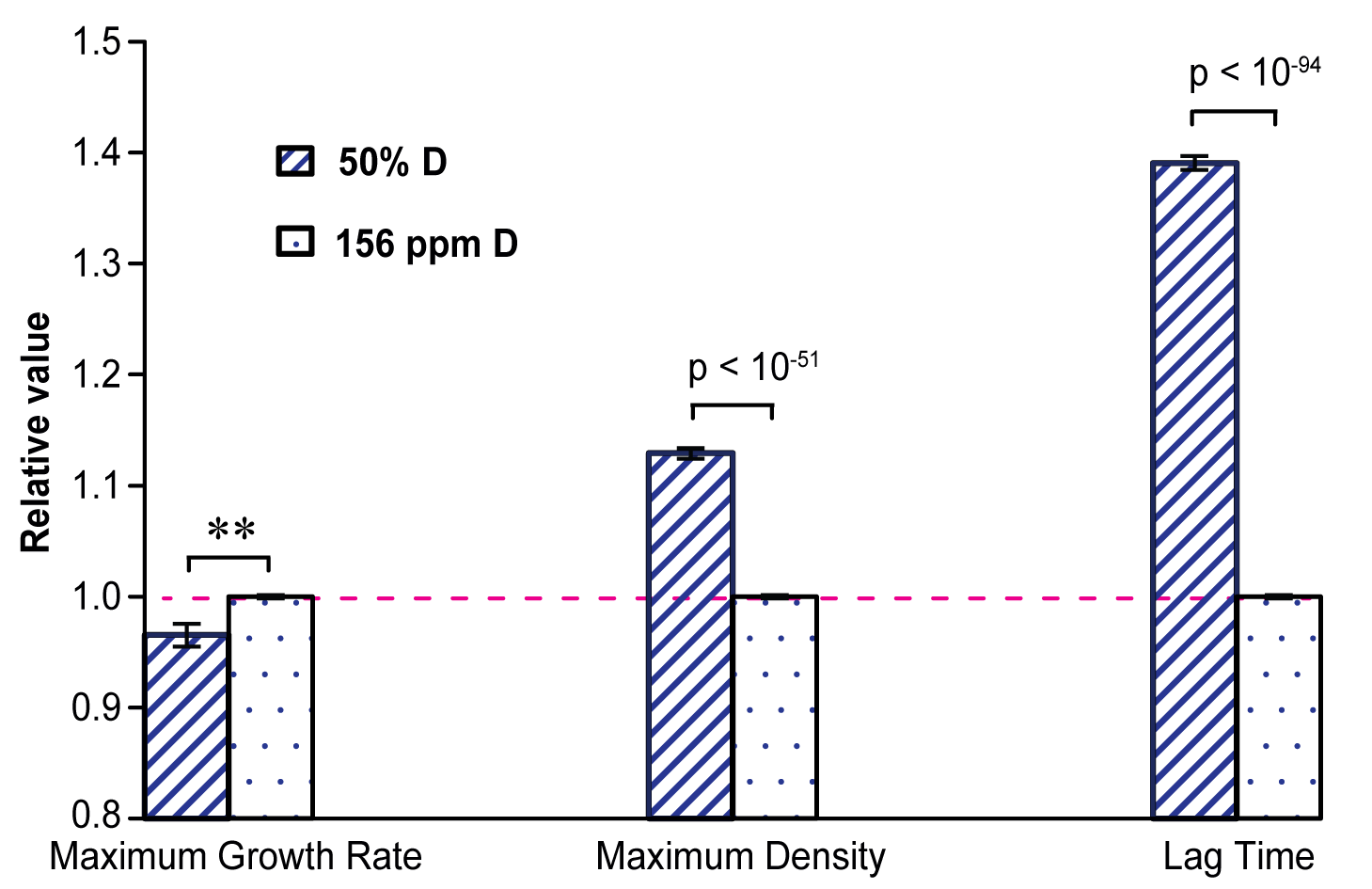

Supplement: Figure S2 — Maximum growth rate, maximum density and lag time of E. coli grown in M9 minimal media with deuterium content of 50% normalized by that at normal deuterium content of 156 ppm. ** is equivalent to p<0.005. (TIF) [file pone.0102071.s002.tif]

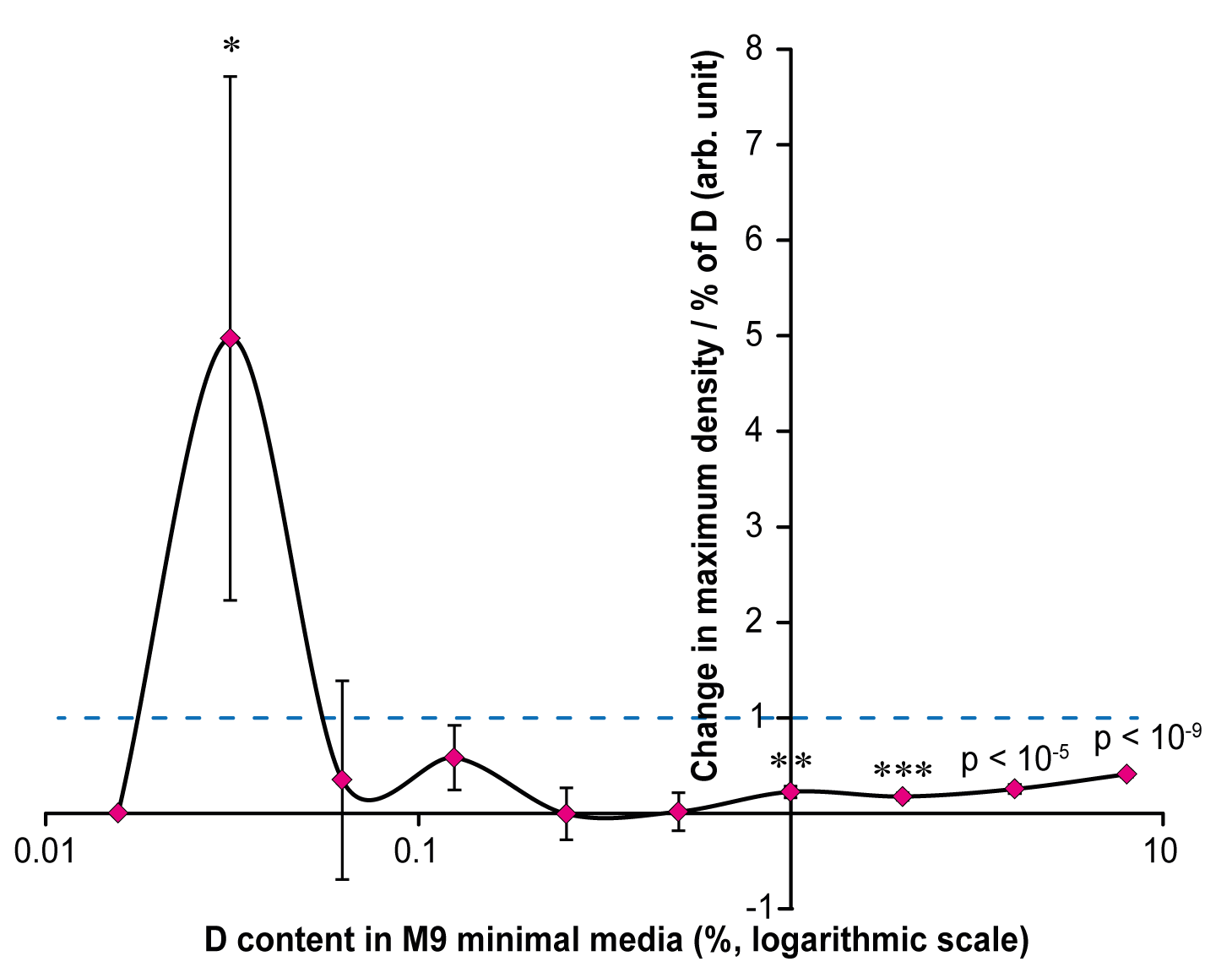

Supplement: Figure S3 — Change of maximum density per percentage of deuterium for E. coli grown in M9 minimal media with content of deuterium from 156 ppm (terrestrial value) to 8%. * is equivalent to p<0.05, ** is equivalent to p<0.005, etc. (TIF) [file pone.0102071.s003.tif]

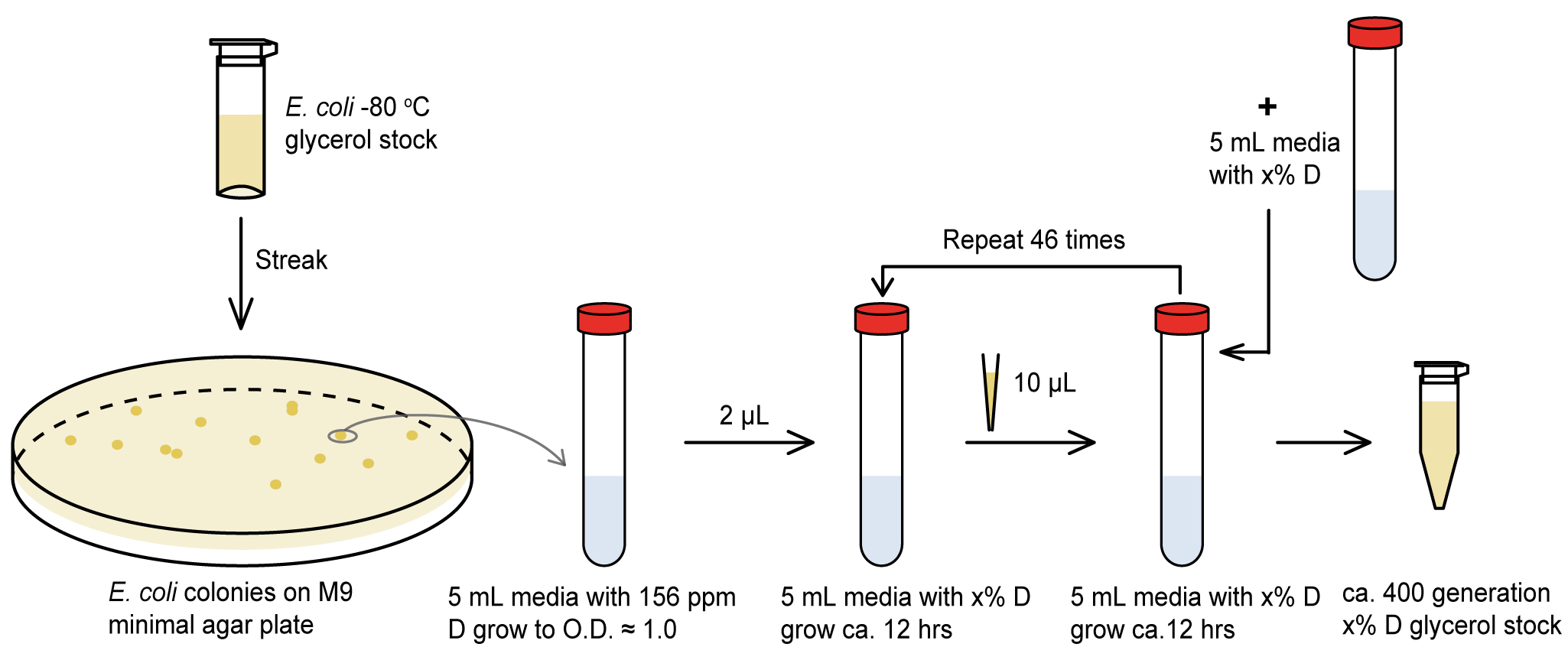

Supplement: Figure S4 — Workflow of adapting the bacteria to growth media. (TIF) [file pone.0102071.s004.tif]

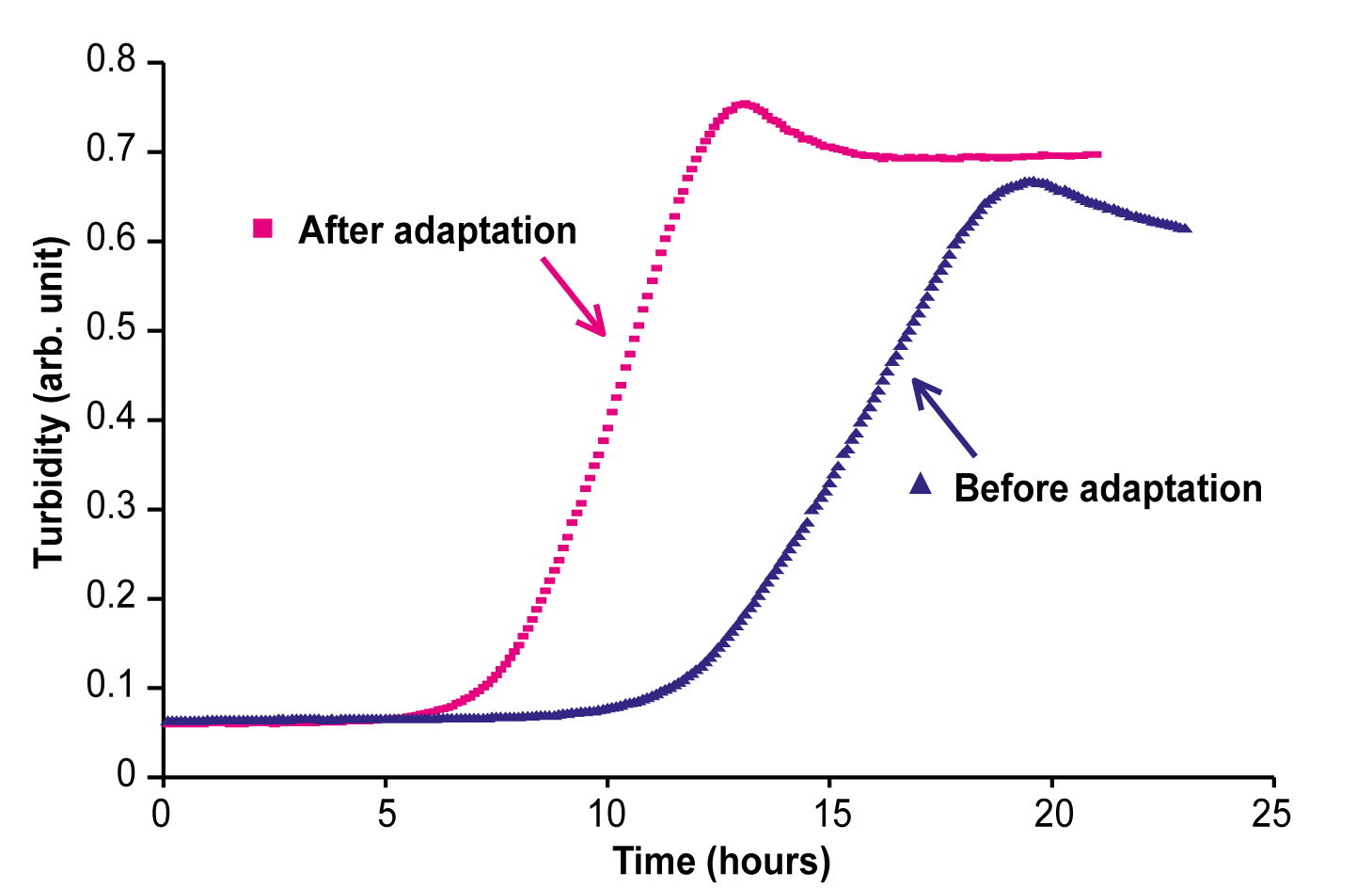

Supplement: Figure S5 — Growth curves of E. coli grown in minimal media with 156 ppm of D before and after adaptation. (TIF) [file pone.0102071.s005.tif]
